# The Use of Point-of-Care Hemoglobin Measurements in an Elderly Population with Hematological Disorders and Anemia

**DOI:** 10.3390/hematolrep18040045

**Published:** 2026-06-30

**Authors:** Ittai Appel, Liat Dizengoff, Nili Stein, Regina Draliuk, Alla Kravits, Shoshan Perek, Amir Warwar, Ibrahim Zoubi, Marwa Naamneh, Adi Kibari, Mouna Ballan-Haj, Olga Valkovsky, Elena Mishchenko, Meir Preis

**Affiliations:** 1 Ruth and Bruce Rappaport Faculty of Medicine, Technion-Israel Institute of Technology, Haifa 3109601, Israel; 2 Institute of Hematology, Lady Davis Carmel Medical Center, Haifa 3436212, Israel; 3 Department of Epidemiology, Lady Davis Carmel Medical Center, Haifa 3436212, Israel

**Keywords:** point-of-care, hemoglobin, blood transfusion

## Abstract

**Background:** Patients with severe chronic anemia often require frequent blood transfusions. Many are elderly with comorbidities and limited mobility, making regular hospital visits burdensome. In some cases, patients may receive transfusions despite hemoglobin levels being above the clinical threshold due to logistical challenges, leading to unnecessary exposure to risks, inefficient use of blood units, and resource strain. This study aims to evaluate the use of point-of-care (POC) hemoglobin measurements under controlled outpatient clinic conditions, as an initial step toward potential future home-based monitoring by the patients or their caregivers, with the goal of optimizing transfusion timing, aiming to reduce unnecessary hospital visits while maintaining patient safety. **Methods:** A total of 127 patients from a hemato-oncology outpatient clinic at Carmel Medical Center were evaluated using a nurse-operated POC device to sample capillary blood, with 236 paired measurements concurrently analyzed via venous blood in the laboratory. Demographic and clinical data were assessed to evaluate factors associated with agreement between POC and laboratory measurements. Statistical analysis included Bland–Altman plots and Pearson correlation coefficients. **Results:** The POC device showed a moderate correlation with laboratory results (r = 0.73, *p* < 0.001), with a mean difference of 1.20 g/dL (SD = 1.94 g/dL) but wide limits of agreement (−3.20 to 5.50 g/dL). No significant differences were observed across demographic or clinical subgroups. Notably, all 156 paired measurements with POC-measured hemoglobin >7 g/dL were confirmed by laboratory testing. **Conclusions:** Although POC hemoglobin devices are not suitable as standalone tools for routine monitoring of chronic anemia, the high negative predictive value observed at the 7 g/dL threshold suggests that they may be useful for ruling out severe anemia. If validated in larger multicenter and home-use studies, POC Hb devices might contribute to reducing unnecessary hospital visits and transfusions.

## 1. Introduction

Anemia exists when circulating red blood cells are insufficient to meet physiological oxygen-carrying needs, as defined by the World Health Organization (WHO) [1].

Anemia might present symptomatically, for example with fatigue, dizziness, exertional breathlessness, palpitations, pale skin, exacerbations of cardiac failure, and angina, or be detected incidentally during routine screening or as part of an evaluation of almost any medical condition [1]. Anemia is associated with poor health outcomes and increased morbidity and mortality [2]. The standard method for assessing RBC levels is a complete blood count (CBC), with hemoglobin (Hb) concentration serving as the primary indicator [3]. Hb thresholds for diagnosing anemia vary among different patient populations and were recently updated by the WHO in 2024 to reflect these variations more accurately [1].

In 2021, anemia had a global prevalence of 24.3% across all age groups [2], highlighting its widespread impact on public health. Approximately 17% of individuals over the age of 65 are affected by anemia [4], underscoring its significant prevalence in the elderly population.

Some patients, particularly those with severe chronic anemia, require frequent blood transfusions. This is typically necessary when Hb concentration falls below 7 g/dL [5], requiring blood transfusion. In Israel, blood transfusions are administered in hospitals [6]. Most patients suffering from chronic anemia are elderly individuals with concomitant medical conditions, often facing mobility limitations [7]. For these patients, frequent hospital visits for blood transfusions, including the waiting time, duration, and discharge process, can be burdensome. The repeated hospital visits not only pose physical and emotional challenges but also increase the risk of acquiring infections [8]. This complex situation places significant strain on both the patients and their caregivers [9].

When a patient arrives at the hospital for a blood transfusion, a CBC is typically performed to measure the Hb concentration in the blood [10]. This test helps assess whether the patient requires a transfusion based on their current Hb level, ensuring appropriate treatment decisions are made. Unfortunately, there may be cases where a patient arrives at the hospital with a Hb level above the threshold for transfusion but still receives one due to difficulties in mobility. In such situations, the patient may be exposed to unnecessary side effects from the transfusion, without a significant improvement in their condition. Additionally, this leads to the waste of blood units, as well as time and resources that could have been used more effectively elsewhere [11]. If a patient’s Hb concentration exceeds the transfusion threshold, and they do not receive the blood unit, this can lead to frustration and disappointment for both the patient and their family and wasted time and resources for both the patient and healthcare providers. In some cases, patients are sent to the emergency room by community physicians to receive blood transfusions without prior planning. This urgent need can sometimes result in inappropriate treatment within the emergency department [10] as well as delays and inefficiencies.

Therefore, our proposed study aims to evaluate the use of a point-of-care (POC) device for Hb measurements under controlled outpatient clinic conditions. Importantly, the present study was designed as an initial, nurse-operated validation of a POC Hb device in a hemato-oncology outpatient clinic under supervised conditions, rather than as an evaluation of patient- or caregiver-operated home monitoring. As a prerequisite for any future evaluation of home-based monitoring, the goal of the present study was to determine whether POC Hb measurements may have potential utility in supporting transfusion-related clinical assessment while maintaining patient safety.

## 2. Materials and Methods

A total of 127 patients attending the hemato-oncology outpatient clinic at Carmel Medical Center were recruited, with some undergoing repeated evaluations across multiple visits. The study was approved by the local IRB committee, and all subjects who participated in the study signed an informed consent form.

In this study, we used the “Hemochroma Plus system (FPRR016; Boditech Med Inc., Chuncheon-si, Republic of Korea)”, a portable in vitro diagnostic device intended for quantification of total Hb concentration in human blood, which has been approved by the U.S. Food and Drug Administration (FDA) [12]. The device uses 15 µL of capillary or venous whole blood, and Hb concentration is determined using spectral photometry (detection range: 0–17 g/dL). As an initial step toward potential home-based Hb monitoring, and in order to evaluate the device’s performance under controlled conditions, an initial validation protocol was developed and implemented as a nurse-operated protocol within the hemato-oncology outpatient clinic. In this phase, capillary blood samples were collected during routine outpatient clinic visits using the second drop of blood following capillary puncture. All POC measurements were performed by the same two trained outpatient hemato-oncology registered nurses throughout the study in order to minimize operator-related variability. Device-specific training was provided by a representative of the manufacturer prior to study initiation. Calibration and quality control procedures were performed throughout the study period according to the manufacturer’s instructions. Concurrent venous blood samples were analyzed using standard laboratory methods, resulting in 236 paired blood samples. The reference laboratory method was performed using the ADVIA hematology analyzer, in which Hb concentration is determined photometrically using the cyanmethemoglobin method. This analyzer served as the reference method for comparison with all POC hemoglobin measurements throughout the study.

For each study assessment, patients underwent capillary fingerstick sampling for POC Hb measurement, followed by concurrent venous blood collection for central laboratory analysis. Clinical decisions regarding blood transfusion were based on laboratory hemoglobin results and clinical assessment rather than on POC measurements.

**Figure 1:**
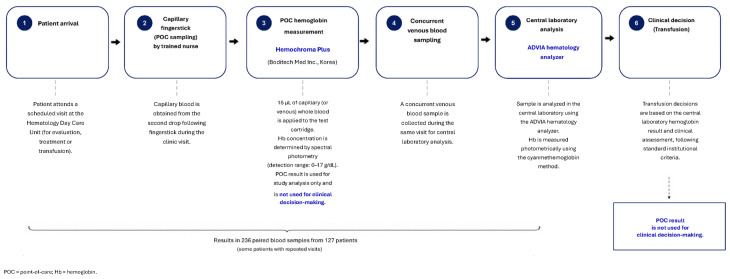
A schematic representation of the nurse-operated clinical workflow used in the present validation study, including patient arrival, capillary fingerstick sampling performed by the same two trained hemato-oncology nurses, point-of-care hemoglobin measurement using the Hemochroma Plus device, concurrent venous blood sampling for central laboratory analysis, and clinical decision-making. Transfusion decisions were based on laboratory hemoglobin results and clinical assessment rather than on point-of-care measurements. The figure illustrates the position of the POC device within the clinical care pathway. Blue text is used to highlight key study information.

A schematic overview of the nurse-operated POC protocol and its position within the clinical care pathway is presented in Figure 1.

To evaluate factors that might be associated with agreement between POC and laboratory measurements, demographic and clinical data—including age, sex, tobacco usage, diagnoses, and medications—were extracted from medical records. Statistical analysis was conducted using Bland–Altman plots and Pearson correlation coefficients, employing IBM SPSS Statistics software (version 28.0, IBM, Armonk, NY, USA).

## 3. Results

During the study period, a total of 236 paired measurements were collected from 127 patients. The mean age of the patients was 69.9 years (standard deviation [SD] = 14.9). Of the 127 patients included in the study, 73 (57.5%) were male. 20 (15.7%) of the patients were identified as current smokers. (see Table S1, Supplementary Materials). Vital signs, including systolic and diastolic blood pressure, pulse rate, and body temperature, were measured, with mean values falling within the normal physiological range. Similarly, the mean left ventricular ejection fraction was within normal limits (see Table S2, Supplementary Materials).Dyslipidemia was the most common comorbidity, affecting 68 (53.5%) patients, followed closely by hypertension, which was present in 63 (49.6%) patients, while diabetes mellitus was diagnosed in 39 patients (30.7%) (see Table S3, Supplementary Materials).

Regarding hematologic conditions, 39 patients (30.7%) were diagnosed with anemia. Multiple myeloma was identified in 46 patients (36.2%), leukemia in 9 patients (7.1%), and lymphoma in 32 patients (25.2%) (Table S3).

Among the analyzed paired measurements, the most common medication exposures were statins (94, 39.8%), beta-1 blockers (92, 39.0%), and non-steroidal anti-inflammatory drugs (NSAIDs) (86, 36.4%) (see Table S4, Supplementary Materials).

Among the analyzed paired measurements, Packed red blood cells (pRBCs) and/or Venofer (treatment A) was administered in 97 measurements (41.1%), Chemotherapy and/or Biological therapy (treatment B) in 135 measurements (57.2%), and Phlebotomy (treatment C) in 4 measurements (1.7%). When both treatment A and treatment B were administered during the same visit, the measurement was classified as treatment A (Table S4).

**Figure 2:**
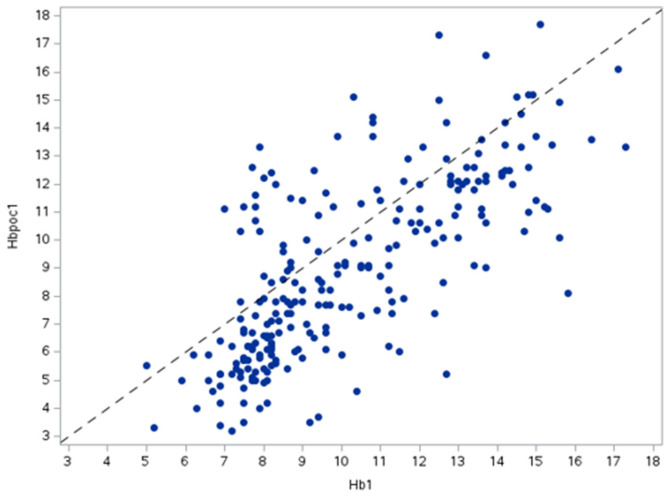
Correlation between point-of-care hemoglobin (HbPOC1) and laboratory hemoglobin (Hb1) measurements. The solid diagonal line represents the line of identity (y = x). A moderate positive correlation was observed (r = 0.73, *p* < 0.001; *n* = 236). Each blue dot represents one paired laboratory and point-of-care hemoglobin measurement.

Hemoglobin levels among laboratory measurements had a mean of 10.14 g/dL (SD = 2.67), ranging from 4.99 to 17.30 g/dL. Point-of-care hemoglobin measurements demonstrated a lower mean value of 8.95 g/dL (SD = 3.20), with a range of 3.20 to 17.70 g/dL (Table S2). When categorizing paired measurements into two groups based on the discrepancy between hemoglobin measurements obtained from the point-of-care (POC) device and the laboratory, only 27 paired measurements (11.4%) exhibited a difference within ±0.5 g/dL (see Table S5, Supplementary Materials). Additional subgroup analysis was performed for paired measurements with laboratory Hb values below 10 g/dL. In this subgroup, 14 of 133 paired measurements (10.5%) demonstrated a difference within ±0.5 g/dL between POC and laboratory Hb measurements (see Table S6, Supplementary Materials). When using a threshold of ±1 g/dL to divide the paired measurements, the agreement improved slightly, with 62 paired measurements (26.3%) falling within this range (see Table S7, Supplementary Materials). The correlation analysis demonstrated a moderate correlation between laboratory hemoglobin measurements (Hb1) and point-of-care (HbPOC1) measurements (r = 0.73, *p* < 0.001) (Figure 2).

**Figure 3:**
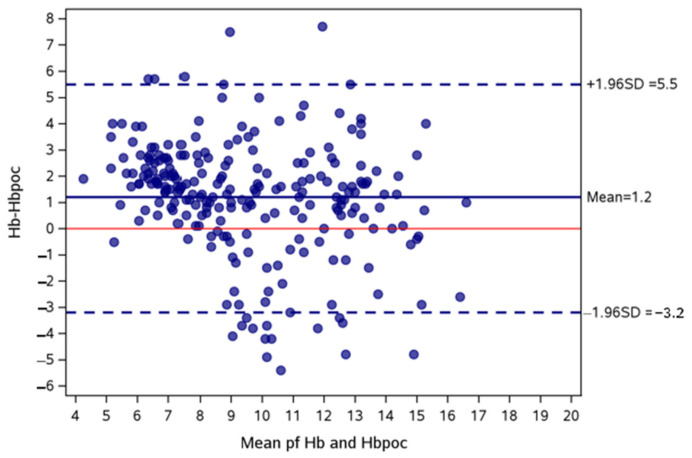
Bland–Altman plot comparing laboratory hemoglobin measurements (Hb1) and point-of-care hemoglobin (HbPOC1) measurements. The mean difference between methods is 1.20 g/dL (SD = 1.94 g/dL), indicating that laboratory values tend to be higher on average. Limits of agreement range from −3.20 to 5.50 g/dL, demonstrating substantial variability between the two methods. The red horizontal line represents zero difference between laboratory and point-of-care hemoglobin measurements.

However, Bland–Altman analysis revealed a mean difference of 1.20 g/dL (SD = 1.94 g/dL), with wide limits of agreement (−3.20 to 5.50 g/dL), indicating substantial variability (Figure 3).

Notably, no specific hemoglobin range exhibited consistently strong agreement between the two methods. To further evaluate the potential relationship between hemoglobin levels and the discrepancy between laboratory-measured hemoglobin and point-of-care device results, paired measurements were stratified into quartiles based on their laboratory hemoglobin values. Within each quartile, paired measurements were further categorized into two groups: those with a hemoglobin discrepancy exceeding ±1 g/dL and those with a discrepancy within ±1 g/dL. The proportion of paired measurements with discrepancies greater than ±1 g/dL remained consistent across quartiles, ranging from 71.2% to 76.6%. Statistical analysis revealed no significant difference in hemoglobin discrepancies across the quartiles (*p* = 0.873), indicating that the magnitude of discrepancy was not associated with specific hemoglobin ranges (Table 1).

Additionally, the analysis divided paired measurements into two groups based on the difference between hemoglobin values obtained from the laboratory and the point-of-care device: those with a difference of ≤1 g/dL and those with a difference >1 g/dL. No significant differences were found between the groups across the demographic and clinical variables analyzed, as none of the tested parameters demonstrated a statistically significant association with the magnitude of hemoglobin discrepancy (Table 2).

In addition, we examined the potential relationship between the type of hemato-oncological treatment administered at day hospitalization (classified as treatments A, B, or C) and the difference between hemoglobin levels measured in the laboratory and those obtained using the point-of-care device. The analysis, conducted for discrepancies of ≤0.5 g/dL and ≤1 g/dL, yielded *p*-values of 0.662 and 0.090, respectively, indicating no statistically significant association between treatment type and the observed hemoglobin discrepancy (see Table S8, Supplementary Materials).

To further assess the agreement between the two measurement methods in identifying critically low hemoglobin levels (<7 g/dL), a cross-tabulation analysis was performed. The results indicated that among the 80 POC measurements classified by the POC device as having hemoglobin levels below 7 g/dL, only 14 (17.5%) were confirmed to have such levels based on laboratory testing. Notably, all 14 laboratory-confirmed measurements with hemoglobin levels below 7 g/dL were correctly identified by the POC device; however, they represented a small proportion of those classified as below the critical range by the device. The kappa coefficient for this analysis was 0.22, indicating poor agreement. Nevertheless, it is important to highlight that all 156 POC measurements classified by the POC device as having hemoglobin levels above 7 g/dL were indeed confirmed as such by laboratory testing (Table 3).

Additional analysis using a laboratory Hb threshold of 8 g/dL identified 9 false-negative results, while 118 of 127 paired measurements with POC Hb levels ≥8 g/dL were confirmed by laboratory testing (see Table S9, Supplementary Materials).

Finally, a receiver operating characteristic (ROC) curve was constructed to identify a cutoff for agreement between the two measurements. The analysis presented an area under the curve (AUC) of 0.523 (95% CI: 0.438–0.607; *p* = 0.596) (see Figure S1, Supplementary Materials).

## 4. Discussion

The primary objective of this study was to evaluate whether a POC Hb device could potentially support the monitoring of chronic transfusion recipients by helping identify patients who may not require immediate clinical evaluation, while facilitating timely assessment of those who may require transfusion. To this end, we performed an initial validation study assessing the agreement between POC and laboratory hemoglobin measurements under controlled clinical conditions.

PRBC transfusions are complex, costly, time-intensive procedures that require thorough medical evaluation, blood sample collection, laboratory analysis, crossmatching, transfusion administration, and post-transfusion hemoglobin reassessment [10]. Despite the significant logistical and clinical burden associated with chronic transfusions, limited research has been conducted in this area, whereas substantial literature exists on the use of POC Hb devices in emergency transfusions [10]. A relevant example of how POC testing for a hematological factor can alter clinical decisions is seen in a study using a POC device to measure coagulation function in stroke patients receiving anticoagulants. By assessing INR levels, the device helps determine if these patients are eligible for stroke treatment [13]. This testing can alter treatment plans, allowing patients previously excluded due to anticoagulant use to receive appropriate stroke care if their INR is within a safe range. Similarly, a study evaluating non-invasive Hb monitoring (SpHb) in neonates demonstrated strong correlation and agreement with invasive venous sampling [14]. The authors proposed that such technology may help reduce iatrogenic anemia by minimizing the need for frequent blood draws [14]. While the population and measurement method differ from our study, these findings underscore the broader potential of alternative Hb assessment tools to improve patient care, pending further validation.

Our findings indicate that while POC Hb measurements exhibit a moderate correlation with central laboratory results, they demonstrate substantial variability, as reflected in the large SD and poor agreement between the two methods. Despite evaluating multiple demographic, clinical, and treatment-related variables, we were unable to identify a subgroup in which agreement between POC and laboratory measurements was substantially improved. However, at the critical transfusion threshold (Hb = 7 g/dL), the POC device demonstrated a high negative predictive value (NPV), as evidenced by the Bland–Altman analysis and cross-tabulation results. A supplementary subgroup analysis of paired measurements with laboratory Hb values below 8 g/dL yielded an NPV of 92.9%. These findings suggest that while POC Hb devices are not suitable for routine monitoring of chronic anemia, their potential clinical utility may differ depending on the direction of interpretation. High POC Hb values may help rule out severe anemia in settings similar to those evaluated in the present study, although this should be interpreted with caution due to the observed variability between methods. In contrast, low POC Hb values demonstrated limited rule-in utility because of the high frequency of false-positive low results and therefore should not be interpreted as evidence of severe anemia without confirmation by central laboratory testing.

At present, the findings of this study do not support the use of the device as a standalone tool for transfusion-related decision-making. Although moderate correlation was observed between POC and laboratory hemoglobin measurements, agreement between methods was limited, with wide limits of agreement and a low positive predictive value at clinically relevant transfusion thresholds. Nevertheless, the high negative predictive value observed at clinically relevant hemoglobin thresholds suggests that further investigation of its potential role as a rule-out tool may be warranted. Our data justify further evaluation of POC Hb as a potential rule-out tool, but they do not support replacing laboratory testing for transfusion decisions. If validated in larger multicenter and home-use studies, POC Hb devices might contribute to reducing unnecessary hospital visits and transfusions.

This study has several strengths, including a relatively large number of paired POC–laboratory hemoglobin measurements obtained from a clinically relevant hemato-oncology population frequently evaluated for anemia and transfusion-related care. In addition, all POC measurements were performed by trained nurses using a standardized protocol, minimizing operator-related variability. The availability of detailed demographic, clinical, and treatment-related data, including hemato-oncological treatment protocols, enabled a comprehensive evaluation of potential factors associated with agreement between POC and laboratory measurements.

Nevertheless, several limitations should be acknowledged. Although the study population was diverse, all participants were recruited from a single medical center, which may limit generalizability. In addition, some clinically relevant subgroups, such as patients undergoing phlebotomy, were represented by small numbers, limiting subgroup-specific analyses. Most notably, although the long-term motivation of this work relates to potential home-based monitoring, all POC measurements in the present study were performed by trained nurses in a controlled clinical setting. Accordingly, the present findings should be interpreted as an initial validation step conducted under supervised conditions rather than as evidence of patient- or caregiver-operated home testing. Therefore, the accuracy, feasibility, and safety of patient- or caregiver-operated home use remain unknown and require dedicated evaluation.

Future studies should validate these findings in larger multicenter cohorts and explore whether incorporating additional hematologic parameters, such as RDW (Red Blood Cell Distribution Width), may provide complementary information for transfusion-related assessment. In addition, prospective studies should evaluate the real-world performance of patient- or caregiver-operated POC Hb monitoring in home settings. Such studies may help determine whether the potential rule-out utility suggested by the present findings can be maintained when testing is performed by patients or caregivers outside the clinical environment. If validated in larger multicenter and home-use studies, POC Hb devices might contribute to reducing unnecessary hospital visits and transfusions. However, the present findings justify further evaluation as a potential rule-out tool and do not currently support replacing laboratory testing for transfusion decisions.

## 5. Conclusions

In conclusion, POC hemoglobin measurements demonstrated only moderate correlation and limited agreement with central laboratory results. At present, the device cannot be considered a standalone tool for routine monitoring of chronic anemia or transfusion-related decision-making. Furthermore, no demographic, clinical, or treatment-related subgroup demonstrated substantially improved agreement. However, the high negative predictive value observed at the 7 g/dL threshold suggests that high POC Hb values may have potential utility for ruling out severe anemia, as observed under the controlled clinical conditions evaluated in the present study. Given the substantial variability observed between methods, low POC Hb values should always be confirmed by laboratory testing. Further studies are needed to determine whether these findings can be reproduced in patient- or caregiver-operated home settings. Although the present findings do not currently support replacing laboratory testing for transfusion decisions, they do justify further evaluation of POC Hb as a potential rule-out tool.

## Figures and Tables

**Table 1:** Agreement between laboratory hemoglobin and point-of-care measurements across hemoglobin quartiles. Paired measurements were stratified into quartiles based on laboratory hemoglobin (Hb) levels and categorized by the magnitude of discrepancy between Hb and HbPOC measurements: either within ±1 g/dL or exceeding ±1 g/dL. The proportion of paired measurements with discrepancies > ±1 g/dL remained consistent across quartiles (range: 71.2–76.6%). No statistically significant difference in discrepancy rates was observed across quartiles (*p* = 0.873).

Hemoglobin Quartile	ΔHb-HbPOC > ±1 g/dL	ΔHb-HbPOC ≤ ±1 g/dL	Total
1st quartile (Hb ≤ 8.00)	*n* = 49 (76.6%)	*n* = 15 (23.4%)	*n* = 64 (100.0%)
2nd quartile (Hb 8.01–9.40)	*n* = 40 (71.4%)	*n* = 16 (28.6%)	*n* = 56 (100.0%)
3rd quartile (Hb 9.41–12.45)	*n* = 43 (75.4%)	*n* = 14 (24.6%)	*n* = 57 (100.0%)
4th quartile (Hb ≥ 12.46)	*n* = 42 (71.2%)	*n* = 17 (28.8%)	*n* = 59 (100.0%)
Total	*n* = 174 (73.7%)	*n* = 62 (26.3%)	*n* = 236 (100.0%)

**Table 2:** Association of demographic, clinical, and treatment-related variables with hemoglobin measurement discrepancy (≤1 g/dL vs. >1 g/dL). Paired measurements were divided into two groups based on whether the absolute difference between point-of-care and laboratory hemoglobin measurements was greater than 1 g/dL or ≤1 g/dL. No significant differences were observed between the groups across demographic, clinical, and treatment-related variables.

Variable	Hb Difference > 1 g/dL (*n* = 174 Paired Measurements)	Hb Difference ≤ 1 g/dL (*n* = 62 Paired Measurements)	*p*-Value
Age	70.8 ± 13.4	68.4 ± 17.0	0.715
Male, *n* (%)	102 (58.6%)	34 (54.8%)	0.605
Hemoglobin	10.10 ± 2.60	10.30 ± 2.80	0.718
Smoking			0.444
Never	111 (63.8%)	44 (72.1%)	
Former	34 (19.5%)	8 (13.1%)	
Current	29 (16.7%)	9 (14.8%)	
Treatment			0.090
A	73 (42.0%)	24 (38.7%)	
B	100 (57.5%)	35 (56.5%)	
C	1 (0.6%)	3 (4.8%)	
BMI	25.3 ± 4.2	25.1 ± 4.3	0.778
Systolic Blood Pressure	121.0 ± 17.6 (*n* = 159)	119.0 ± 16.7 (*n* = 55)	0.324
Diastolic Blood Pressure	62.3 ± 12.6 (*n* = 159)	63.2 ± 11.6 (*n* = 55)	0.994
Peripheral Vascular Disease	2 (1.2%)	3 (3.2%)	0.284
Diabetes Mellitus	58 (33.5%)	22 (36.1%)	0.719
Neuropathy	12 (6.9%)	4 (6.5%)	>0.99
Multiple Myeloma	55 (32.4%)	17 (27.4%)	0.472
Leukemia	25 (14.5%)	7 (11.3%)	0.534
Lymphoma	32 (18.5%)	12 (19.4%)	0.882
Hemoglobinopathy	9 (5.2%)	4 (6.5%)	0.749
Anemia	65 (37.4%)	25 (40.3%)	0.680
Polycythemia	5 (2.9%)	4 (6.5%)	0.249
Bone Marrow Disorder	46 (26.6%)	11 (17.7%)	0.163
Receiving transfusions	77 (44.3%)	23 (37.1%)	0.328
Phlebotomy	2 (1.1%)	0	>0.99
Aranesp Therapy	4 (2.4%)	2 (3.4%)	0.651
Binocrit Therapy	36 (21.4%)	9 (15.3%)	0.306
Anticoagulation Drug Therapy	41 (24.4%)	11 (18.6%)	0.365
Anti-aggregation Drug Therapy	46 (27.4%)	20 (33.9%)	0.343

**Table 3:** Cross-tabulation of point-of-care (POC) hemoglobin measurements and laboratory (Lab)-confirmed hemoglobin below 7 g/dL. The POC device correctly identified all laboratory-confirmed cases of critical anemia, and yielded a negative predictive value (NPV) of 100%. A negative POC result (>7 g/dL) correctly ruled out critical anemia (laboratory Hb < 7 g/dL) in all cases. Overall agreement between methods was limited (kappa = 0.22). *n* = 236 paired measurements.

	Lab Hb < 7 g/dL	Lab Hb ≥ 7 g/dL	Total
POC Hb < 7 g/dL	14 (True Positive)	66 (False Positive)	80
POC Hb ≥ 7 g/dL	0 (False Negative)	156 (True Negative)	156
Total	14	222	236

## Data Availability

The data that support the findings of this study are available from the corresponding author upon reasonable request.

## Supplementary Materials

The following supporting information can be downloaded at: https://www.mdpi.com/article/10.3390/hematolrep18040045/s1. The following supporting information is available: Table S1. Demographics, Table S2. Clinical data, Table S3. Medical conditions, Table S4. Medications and treatments associated with the analyzed paired measurements, Table S5. Distribution of paired measurements by delta laboratory hemoglobin (Lab Hb)–point-of-care hemoglobin (POCHb) using ±0.5 g/dL thresholds, Table S6. Distribution of paired measurements with laboratory Hb values below 10 g/dL by delta laboratory hemoglobin (Lab Hb)–point of care hemoglobin (POCHb) using ±0.5 g/dL threshold, Table S7. Distribution of paired measurements by delta laboratory hemoglobin (Lab Hb)–point-of-care hemoglobin (POCHb) using ±1.0 g/dL thresholds, Table S8. Distribution of the 236 paired measurements by the difference between laboratory hemoglobin (Lab Hb) and point-of-care hemoglobin (POC Hb), using ±0.5 g/dL and ±1.0 g/dL thresholds, stratified by treatment group, Table S9. Cross-tabulation and Figure S1. ROC curve for identifying a cutoff on point-of-care hemoglobin to predict agreement with laboratory hemoglobin.
