# Supplementary material for: The Use of Point-of-Care Hemoglobin Measurements in an Elderly Population with Hematological Disorders and Anemia
Source: Hematol Rep. 2026 Jun 30;18(4):45. doi: 10.3390/hematolrep18040045 (PMC13398108; doi:10.3390/hematolrep18040045)

hematology reports

# Supplementary Materials

**Table S1. Demographics**. Baseline characteristics derived from 127 unique patients, including demographic distributions (sex, age, ethnicity, smoking status), , and distances from the hospital. Demographic variables are presented per unique patient (N = 127).

| **Variable** | **n (%)** |
|---|---|
| Male | 73 (57.5%) |
| **Female** | **54 (42.5%)** |
| Former smoker | 19 (15.0%) |
| Current smoker | 20 (15.7%) |
| Non-smoker | 87 (68.5%) |
| Age | 69.9 ± 14.9 (18.0–92.0) |
| Distance from hospital among patients living outside Haifa (n=79) | 23.7 ± 14.5 |
| Live in Haifa (n=48) | 48 (37.8%) |
| Ethnicity | |
| Jewish | 39 (30.7%) |
| Arab | 33 (26.0%) |
| Immigrant to Israel (Oleh) | 55 (43.3%) |

**Table S2. Clinical data.** Clinical parameters relevant to hematologic and cardiovascular status. Laboratory and physiological measurements are presented per paired measurement (N = 236) and are reported as mean ± SD with corresponding minimum and maximum values.

| Variable Name | Mean ± SD (Min–Max) | Units | Normal Range |
|---|---|---|---|
| Hemoglobin | 10.14 ± 2.67 (4.99–17.30) | g/dL | Men 13.50-17.50<br>Women 11.50-16.60 |
| Hemoglobin Point-of-Care | 8.95 ± 3.20 (3.20–17.70) | g/dL | Men 13.50-17.50<br>Women 11.50-16.60 |
| Temperature | 36.62 ± 0.22 (36.00–37.60) | °C | 35.00–38.00 |
| Pulse Rate | 79.78 ± 14.37 (47.00–146.00) | beats/min | 60.00–100.00 |
| Systolic Blood Pressure | 120.55 ± 17.34 (79.00–170.00) | mmHg | 90.00–140.00 |
| Diastolic Blood Pressure | 62.96 ± 12.29 (33.00–96.00) | mmHg | 60.00–90.00 |
| Mean Corpuscular Hemoglobin (MCH) | 29.98 ± 3.26 (20.30–38.70) | pg | 26.00–34.00 |
| Mean Corpuscular Hemoglobin Concentration (MCHC) | 32.58 ± 1.17 (29.10–36.00) | g/dL | 31.00–37.00 |

 https://doi.org/10.3390/xxxxx

| | | | |
|---|---|---|---|
| Glucose | 126.50 ± 58.74 (55.00–415.00) | mg/dL | 70.00–100.00 |
| Urea | 46.40 ± 23.20 (13–138) | mg/dL | 17.00–43.00 |
| Creatinine | 1.06 ± 0.48 (0.45–3.74) | mg/dL | Men 0.67–1.17<br>Women 0.51–0.95 |
| Estimated Glomerular Filtration Rate | 72.28 ± 36.11 (13.00–229.00) | mL/min/1.73m[2] | >60.00 |
| Fibrinogen | 355.25 ± 131.75 (110.00–956.00) | mg/dL | 200.00–400.00 |
| Prothrombin Time (PT) | 24.27 ± 104.99 (9.90–1031.00) | seconds | 11.00–13.5.00 |
| Partial Thromboplastin Time (PTT) | 29.94 ± 5.88 (21.00–53.90) | seconds | 25.00–35.00 |
| Left Ventricular Ejection Fraction (LVEF) | 58.18 ± 9.55 (0.00–75.00) | % | 50.00–70.00 |

**Table S3. Medical conditions.** Comorbidity profiles are presented per unique patient (N = 127).

| **Category** | **Variable** | **n (%)** |
|---|---|---|
| Cardiovascular Diseases | Hypertension | 63 (49.6%) |
| | Dyslipidemia | 68 (53.5%) |
| | Chronic Heart Failure (CHF) | 12 (9.4%) |
| | Left Ventricular Hypertrophy | 17 (13.4%) |
| | Myocardial Infarction (MI) | 4 (3.1%) |
| | Peripheral Vascular Disease (PVD) | 3 (2.4%) |
| Endocrine & Metabolic Disorders | Diabetes Mellitus (DM) | 39 (30.7%) |
| | Hypothyroidism | 10 (7.9%) |
| Hematological & Oncological Conditions | Anemia | 39 (30.7%) |
| | Multiple Myeloma | 46 (36.2%) |
| | Lymphoma | 32 (25.2%) |
| | Leukemia | 9 (7.1%) |
| | Bone Marrow Disorders | 20 (15.7%) |
| | Hypercoagulable State | 7 (5.5%) |
| | Thrombocytopenia | 3 (2.4%) |
| | Solid Malignancy | 22 (17.3%) |
| Other Conditions | Neuropathy | 9 (7.1%) |
| | Autoimmune Diseases | 10 (7.9%) |
| | Chronic Obstructive Pulmonary Disease (COPD) | 12 (9.4%) |

**Table S4. Medications and treatments associated with the analyzed paired measurements.** Frequencies and proportions of medications and treatment exposures recorded during the analyzed paired measurements, grouped by pharmacologic categories. These include hematologic/oncologic therapies, cardiovascular and antihypertensive agents, anti-inflammatory medications, and treatment protocols administered during day hospitalization.

| Drug Category | Variable | n (%) |
|---|---|---|
| Hematologic and Oncologic Therapies | Jakavi | 3 (1.3%) |
| | Aranesp | 6 (2.5%) |
| | Binocrit | 45 (19.1%) |
| | Tevagrastim | 42 (17.8%) |
| | Revolade | 8 (3.4%) |
| | Hexakapron | 13 (5.5%) |
| Cardiovascular and Vasoactive Drugs | Statins | 94 (39.8%) |
| | Anticoagulation Therapy | 52 (22.0%) |
| | Anti-Aggregation Therapy | 66 (28.0%) |
| | Antiarrhythmic Drugs | 9 (3.8%) |
| | Alpha-1 Agonists | 10 (4.2%) |
| | Beta-2 Agonists | 14 (5.9%) |
| → Antihypertensive Drugs | ACE Inhibitors | 38 (16.1%) |
| | ARBs | 52 (22.0%) |
| | CCBs | 40 (16.9%) |
| | Beta-1 Blockers | 92 (39.0%) |
| | Alpha-1 Blockers | 37 (15.7%) |
| | Mineralocorticoid Receptor Blockers | 16 (6.8%) |
| | Diuretics | 38 (16.1%) |
| Anti-inflammatory Drugs | NSAIDs | 86 (36.4%) |
| | DMARDs | 3 (1.3%) |
| | Corticosteroids | 78 (33.1%) |
| Day Hospitalization Protocol | Treatment A (pRBCs and/or Venofer) | 97 (41.1%) |
| | Treatment B (Chemotherapy and/or Biological therapy) | 135 (57.2%) |
| | Treatment C (Phlebotomy) | 4 (1.7%) |

N=236 paired measurements.

**Table S5. Distribution of paired measurements by delta laboratory hemoglobin (Lab Hb)–point-of-care hemoglobin (POCHb) using ±0.5 g/dL thresholds.**

Paired measurements categorized by the absolute difference between POCHb and laboratory hemoglobin values, using a ±0.5 g/dL threshold as a marker of agreement. The majority (88.6%) of measurements showed differences exceeding ±0.5 g/dL. N=236 paired measurements.

| Delta Lab Hb - POCHb Group | Frequency | Percent | Valid Percent |
|---|---|---|---|
| > 0.5 or < -0.5 g/dL | 209 | 88.6 | 88.6 |
| ≥ -0.5 and ≤ 0.5 g/dL | 27 | 11.4 | 11.4 |
| Total | 236 | 100.0 | 100.0 |

**Table S6.** Distribution of paired measurements with laboratory Hb values below 10 g/dL by delta laboratory hemoglobin (Lab Hb)–point of care hemoglobin (POCHb) using ±0.5 g/dL threshold.

Table S6. Paired measurements with laboratory Hb values below 10 g/dL categorized by the absolute difference between POCHb and laboratory hemoglobin values, using a ±0.5 g/dL threshold. The majority (89.5%) of measurements showed differences exceeding ±0.5 g/dL, while only 10.5% fell within this range.

| Delta Lab Hb - POCHb Group | Frequency | Percent | Valid Percent |
|---|---|---|---|
| > 0.5 or < -0.5 g/dL | 119 | 89.5 | 89.5 |
| ≥ -0.5 and ≤ 0.5 g/dL | 14 | 10.5 | 10.5 |
| Total | 133 | 100.0 | 100.0 |

N=133 out of 236 paired measurements.

**Table S7. Distribution of paired measurements by delta laboratory hemoglobin (Lab Hb)–point-of-care hemoglobin (POCHb) using a ±1.0 g/dL threshold.** Paired measurements categorized by the absolute difference between POCHb and laboratory hemoglobin values. A distribution using a ±1.0 g/dL threshold is presented, showing a larger proportion of paired measurements (26.3%) fell within this broader range of agreement. N = 236 paired measurements.

| Delta Hb-POC Group | Frequency | Percent | Valid Percent |
|---|---|---|---|
| > 1 or < -1 g/dL | 174 | 73.7 | 73.7 |
| ≥ -1 and ≤ 1 g/dL | 62 | 26.3 | 26.3 |
| Total | 236 | 100.0 | 100.0 |

**Table S8.** Distribution of the 236 paired measurements by the difference between laboratory hemoglobin (Lab Hb) and point-of-care hemoglobin (POC Hb), using ±0.5 g/dL and ±1.0 g/dL thresholds, stratified by treatment group.

**Table S8.** Distribution of paired measurements across treatment groups based on the difference between laboratory hemoglobin (Lab Hb) and point-of-care hemoglobin (POC Hb), using ±0.5 g/dL and ±1.0 g/dL thresholds. The majority of paired measurements in each treatment group showed discrepancies greater than these thresholds. No significant difference was observed between treatment groups for the ±0.5 g/dL threshold (P = 0.662), while a trend toward significance was noted for the ±1.0 g/dL threshold (P = 0.090). N=236 paired measurements.

**P = 0.662**

| | **Delta Lab Hb - POCHb > 0.5 or < -0.5** | **Delta Lab Hb – POCHb ≥ -0.5 and ≤ 0.5** | **Total** |
|---|---|---|---|
| **Treatment A** | | | |
| Count | 87 | 10 | 97 |
| Percent within treatment group | 89.7% | 10.3% | 100.0% |
| **Treatment B** | | | |
| Count | 119 | 16 | 135 |
| Percent within treatment group | 88.1% | 11.9% | 100.0% |
| **Treatment C** | | | |
| Count | 3 | 1 | 4 |
| Percent within treatment group | 75.0% | 25.0% | 100.0% |
| **Total** | | | |
| Count | 209 | 27 | 236 |
| Percent within treatment group | 88.6% | 11.4% | 100.0% |

**P=0.090**

| | **Delta Lab Hb - POCHb > 1 or < -1** | **Delta Lab Hb - POCHb ≥ -1 and ≤ 1** | **Total** |
|---|---|---|---|
| **Treatment A** | | | |
| Count | 73 | 24 | 97 |
| Percent within treatment group | 75.3% | 24.7% | 100.0% |
| **Treatment B** | | | |
| Count | 100 | 35 | 135 |
| Percent within treatment group | 74.1% | 25.9% | 100.0% |
| **Treatment C** | | | |
| Count | 1 | 3 | 4 |
| Percent within treatment group | 25.0% | 75.0% | 100.0% |
| **Total** | | | |
| Count | 174 | 62 | 236 |

| | | | |
|---|---|---|---|
| Percent within treatment group | 73.7% | 26.3% | 100.0% |

**Table S9.** Cross-tabulation.

**Table S9**. Cross-tabulation of point-of-care (POC) hemoglobin measurements and laboratory (Lab)-confirmed hemoglobin below 8 g/dL. The POC device yielded a negative predictive value (NPV) of 92.9%. POC result (≥8 g/dL) correctly ruled out laboratory Hb <8 g/dL in 118 of 127 paired measurements.

| | **Lab Hb < 8 g/dL** | **Lab Hb ≥ 8 g/dL** | **Total** |
|---|---|---|---|
| POC Hb < 8 g/dL | 49 (True Positive) | 60 (False Positive) | 109 |
| POC Hb ≥ 8 g/dL | 9 (False Negative) | 118 (True Negative) | 127 |
| Total | 58 | 178 | 236 |

N=236 paired measurements.

**Figure S1.** ROC curve for identifying a cutoff on point-of-care hemoglobin to predict agreement with laboratory hemoglobin.

**Figure S1.** Receiver operating characteristic (ROC) curve assessing the ability of point-of-care hemoglobin to distinguish between cases with and without agreement with laboratory hemoglobin values. The area under the curve (AUC) was 0.523 (95% CI: 0.438–0.607; p = 0.596).

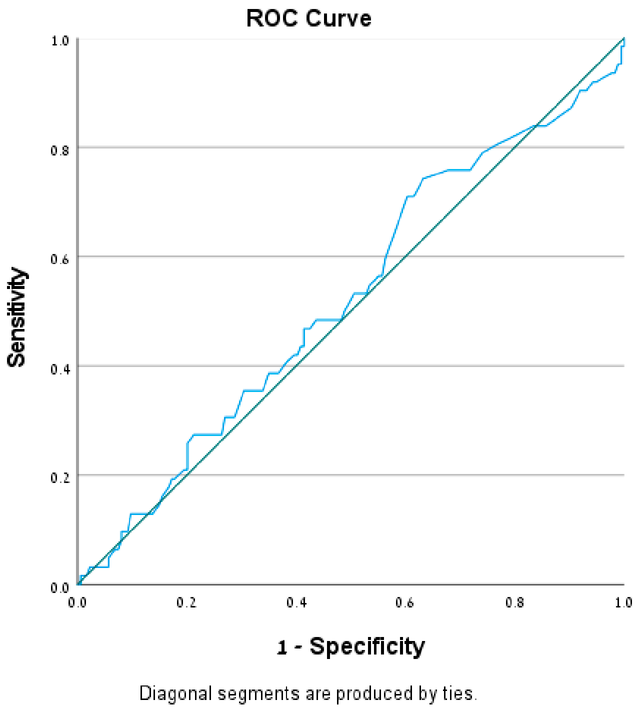

Supplement: Supplementary file 1 [file hematolrep-18-00045-s001.zip › hematolrep-4257555-supplementary.pdf]
